# Cell Line–Dependent Cell Death Pathways Induced by Thymoquinone in Colorectal Cancer Cells

**DOI:** 10.3390/molecules31030512

**Published:** 2026-02-02

**Authors:** Natalia Kurowska, Maria Książek, Paulina Borkowska, Barbara Strzałka-Mrozik

**Affiliations:** 1 Department of Molecular Biology, Faculty of Pharmaceutical Sciences in Sosnowiec, Medical University of Silesia, 41-200 Sosnowiec, Poland; natalka.kurowska@gmail.com (N.K.);; 2 Department of Medical Genetics, Faculty of Pharmaceutical Sciences in Sosnowiec, Medical University of Silesia, 41-200 Sosnowiec, Poland

**Keywords:** thymoquinone, colorectal cancer, cell death pathways, apoptosis, caspase-independent cell death, necrosis, molecular mechanisms, 5-fluorouracil

## Abstract

Colorectal cancer (CRC) remains a leading cause of cancer-related mortality, with resistance to 5-fluorouracil (5-FU) representing a major therapeutic challenge. Thymoquinone (TQ), a bioactive constituent of *Nigella sativa*, exhibits anticancer activity; however, the mechanisms underlying TQ-induced cell death appear to be highly context dependent. This study aimed to characterize cell line-specific death pathways triggered by TQ in colorectal cancer models with distinct molecular backgrounds and differential responsiveness to 5-FU. Human CRC cell lines RKO (5-FU-sensitive) and SW1116 (poorly responsive), along with normal colon epithelial cells (CCD-841CoN), were treated with TQ, 5-FU, or their combination for 24 h. Cell viability, DNA fragmentation, caspase-3/7, -8, and -9 activity, cell death phenotypes, and expression of apoptosis- and necroptosis-related genes were evaluated using MTT assays, ELISA, luminescent assays, flow cytometry, and RT-qPCR. TQ significantly reduced viability in both CRC cell lines while exerting minimal cytotoxicity toward normal cells. In RKO cells, characterized by microsatellite instability (MSI), TQ induced DNA fragmentation, caspase activation, and transcriptional upregulation of pro-apoptotic genes, consistent with apoptosis-associated signaling. In contrast, SW1116 cells, which exhibit chromosomal instability (CIN) and reduced responsiveness to 5-FU, displayed decreased viability accompanied by suppressed caspase activity and predominant features of caspase-independent necrotic cell death. This differential response may be attributed to the CIN phenotype, which has been associated with impaired apoptotic signaling and enhanced tolerance to cytotoxic stress. Combined TQ and 5-FU treatment did not produce synergistic cytotoxicity, as confirmed by Bliss independence analysis, but revealed distinct, cell line-dependent death programs. These findings demonstrate that TQ modulates cell death execution in a molecular context-dependent manner rather than enhancing 5-FU efficacy through pharmacological synergy.

## 1. Introduction

Colorectal cancer (CRC) is one of the most commonly diagnosed malignancies worldwide and remains a leading cause of cancer-related mortality, ranking second globally [1]. A particularly concerning trend is the increasing incidence of early-onset CRC, with approximately 20% of newly diagnosed cases occurring in individuals aged ≤54 years [2,3]. Both non-modifiable factors, including age, family history, genetic predisposition, and inflammatory bowel diseases, and modifiable lifestyle-related determinants, such as obesity, a Western-type diet, low physical activity, smoking, and excessive alcohol consumption, substantially contribute to CRC risk, especially among younger adults [3,4,5,6].

The current standard of care for CRC includes surgical resection, chemotherapy, radiotherapy, and targeted therapies [7,8]. Fluoropyrimidines, primarily 5-fluorouracil (5-FU) and its oral prodrug capecitabine, remain the cornerstone of systemic treatment, either as monotherapy or within combination regimens such as FOLFOX-4 or FOLFIRI [9,10]. However, both intrinsic and acquired resistance to 5-FU are frequently observed and arise from complex genetic, epigenetic, and microenvironmental mechanisms, ultimately contributing to treatment failure and unfavorable clinical outcomes [11,12]. Indeed, the development of resistance to 5-FU represents one of the principal obstacles in the clinical management of CRC and affects the majority of patients with metastatic disease [11].

Resistance to 5-FU is multifactorial and highly heterogeneous [11,13]. It involves alterations in the expression and activity of enzymes responsible for 5-FU metabolism, particularly increased thymidylate synthase activity, which represents the primary molecular target of this drug [14,15,16]. In addition, CRC cells may evade 5-FU-induced cytotoxicity through dysregulation of apoptosis and autophagy, including impairment of p53-dependent signaling and modulation of key survival pathways such as PI3K–AKT and MAPK–ERK [17,18]. Other important mechanisms contributing to resistance include enhanced drug efflux mediated by membrane transporters, adaptive interactions with the tumor microenvironment, and epigenetic reprogramming of cancer cells [11,19,20,21].

Notably, tumors exhibiting chromosomal instability (CIN), such as SW1116 cells, display increased genomic heterogeneity, which promotes the emergence of resistant subclones and facilitates adaptation to cytotoxic stress. This phenotypic plasticity further limits the efficacy of 5-FU–based chemotherapy and contributes to poor therapeutic response [22].

Given the complexity and diversity of resistance mechanisms, there is a critical need to identify novel therapeutic strategies capable of restoring chemosensitivity or bypassing classical resistance pathways. These challenges are further exacerbated by tumor heterogeneity, late-stage diagnosis, and the increasing incidence of CRC among younger patients, for whom optimized therapeutic strategies remain limited [3,23,24]. Collectively, these factors underscore the urgent need for innovative approaches aimed at improving treatment efficacy and overcoming drug resistance.

In recent years, natural bioactive compounds have attracted increasing attention as modulators of anticancer responses due to their pleiotropic mechanisms of action and generally favorable safety profiles [25,26,27].

Thymoquinone (TQ), the principal bioactive constituent of black cumin (*Nigella sativa*) seed oil [28], exhibits antioxidant, anti-inflammatory, immunomodulatory, and anticancer activities mediated through modulation of multiple signaling pathways, including Nrf2, NF-κB, PI3K/AKT, STAT3, p53, and PTEN [29,30,31,32,33,34]. Experimental studies have shown that TQ can inhibit tumor cell proliferation, angiogenesis, and metastatic potential, induce programmed cell death, and protect normal tissues from treatment-related toxicity [29,30,32,33,34]. Owing to its multitarget activity and relatively low toxicity, TQ has been widely investigated as a candidate for combination approaches with conventional chemotherapeutics, particularly in the context of redox regulation and stress-related signaling [35,36,37,38,39].

Most studies evaluating the effects of TQ in colorectal cancer have focused on HCT116, HT29, LoVo, COLO205, and SW480 cell lines, as well as animal and organoid models [40,41,42,43,44,45,46]. In contrast, data regarding the responses of RKO and SW1116 cells remain limited, despite their distinct molecular characteristics and differential sensitivity to chemotherapeutic agents. RKO cells are characterized by microsatellite instability (MSI) and retain wild-type p53, whereas SW1116 cells display chromosomal instability (CIN) [47,48]. Importantly, RKO cells are reported to be relatively sensitive to 5-FU, while SW1116 cells exhibit reduced responsiveness, providing a relevant experimental framework for investigating cell line–dependent responses associated with drug sensitivity and resistance [49]. Comparative analysis of such divergent models enables exploration of context-dependent cellular responses to anticancer agents without assuming a uniform resistance mechanism.

The 24 h time point was selected based on previous reports and preliminary observations indicating that this duration is sufficient to capture early cytotoxic responses and activation of cell death pathways, while still allowing evaluation of downstream execution phases of programmed cell death [50,51,52,53].

Therefore, the present study aimed to investigate cell line-dependent responses to thymoquinone, administered alone or in combination with 5-fluorouracil, in RKO and SW1116 colorectal cancer cell lines, using normal colon epithelial CCD-841CoN cells as a reference. Particular emphasis was placed on the analysis of cell viability, cell death modalities, and transcriptional regulation of apoptosis- and necroptosis-related genes to elucidate the molecular basis of differential cell death pathways triggered by thymoquinone in distinct colorectal cancer models.

## 2. Results

This study examines the effects of thymoquinone, 5-fluorouracil, and their combination on cell viability and cell death-associated responses in colorectal cancer cell lines (RKO and SW1116), as well as in normal colon epithelial cells (CCD-841CoN).

### 2.1. Cell Viability

#### 2.1.1. Cell Viability Following 24 h Exposure to 5-Fluorouracil

**Figure 1:**
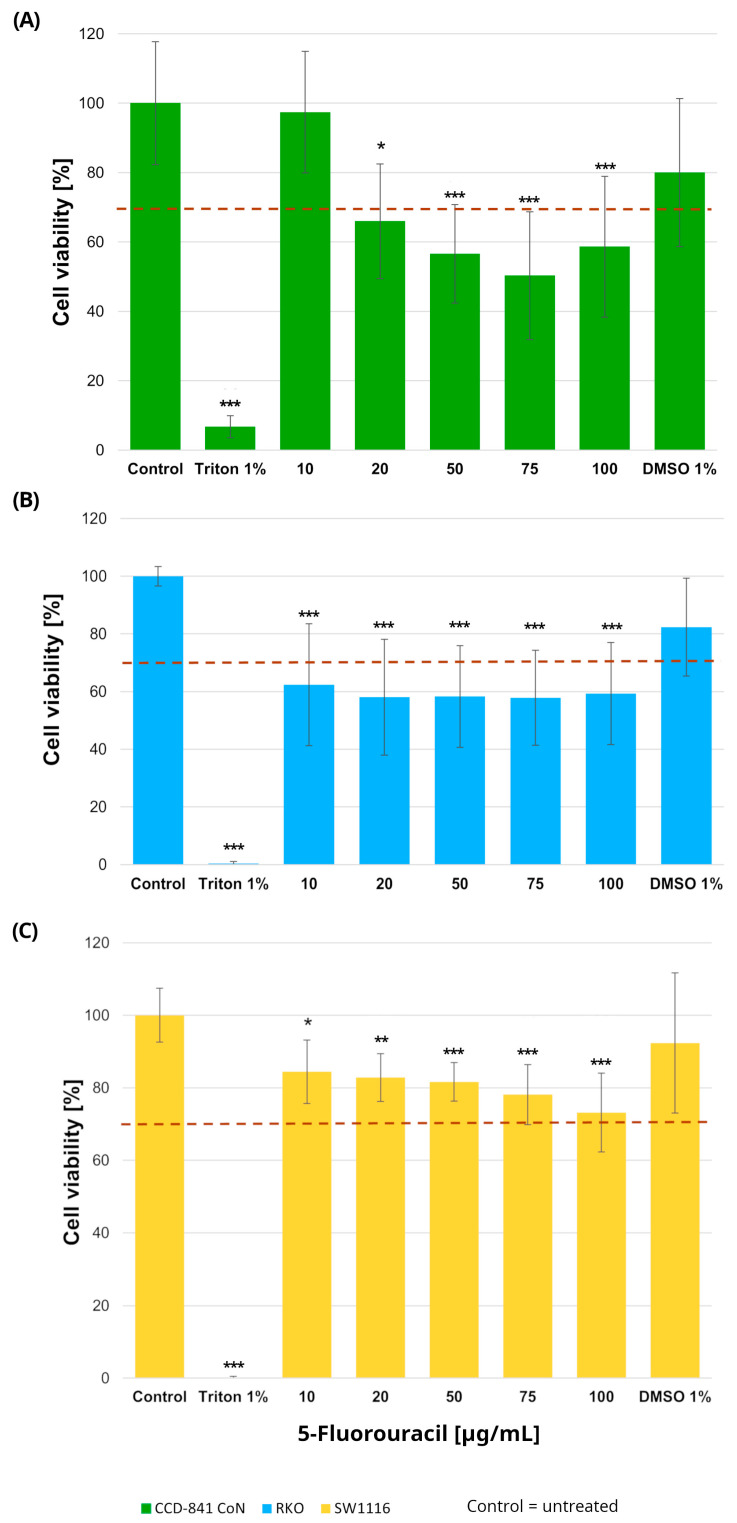
Cell viability of normal colon epithelial cells (CCD-841CoN; (**A**)) and colorectal cancer cell lines RKO (**B**) and SW1116 (**C**) after 24 h exposure to 5-fluorouracil. Cell viability was assessed using the MTT assay and expressed as a percentage of the untreated control (set to 100%). Bars represent mean ± standard deviation (SD). The dashed horizontal line indicates the 70% viability threshold. Untreated cells served as the control, Triton X-100 (1%) was used as a positive cytotoxicity control, and DMSO (1%) served as the vehicle control corresponding to the highest solvent concentration applied. Statistical significance was evaluated using the Kruskal–Wallis one-way ANOVA followed by post hoc pairwise mean-rank comparisons; * *p* < 0.05; ** *p* < 0.01; *** *p* < 0.001 compared with the control. Three independent biological replicates were analyzed. Abbreviations: DMSO, dimethyl sulfoxide.

Normal colon epithelial cells and colorectal cancer cell lines were treated with increasing concentrations of 5-fluorouracil (10–100 µg/mL), resulting in distinct viability profiles across the three cell types (Figure 1). In RKO cells, 5-FU induced a pronounced cytotoxic response, with all tested concentrations (10, 20, 50, 75, and 100 µg/mL) leading to a statistically significant reduction in cell viability compared with untreated controls. Specifically, cell viability decreased to 62 ± 21%, 58 ± 20%, 58 ± 17%, 58 ± 16%, and 59 ± 17% at 10, 20, 50, 75, and 100 µg/mL, respectively (*p* = 0.0005, *p* = 0.00002, *p* = 0.00003, *p* = 0.00003, and *p* = 0.00006).

In contrast, SW1116 cells exhibited a markedly higher tolerance to 5-FU. Although statistically significant differences relative to control were observed at the same concentration range, cell viability in this cell line did not fall below the 70% cytotoxicity threshold at any tested dose, indicating limited sensitivity to 5-FU under the applied conditions.

In the normal colon epithelial CCD-841CoN cell line, cell viability decreased significantly at all tested concentrations except 10 µg/mL, which was the only dose that maintained viability above the 70% threshold. Specifically, cell viability was reduced to 66 ± 16% at 20 µg/mL (*p* = 0.015), 57 ± 14% at 50 µg/mL (*p* = 0.000017), 50 ± 18% at 75 µg/mL (*p* < 1 × 10^−6^), and 59 ± 20% at 100 µg/mL (*p* = 0.0001). Accordingly, this concentration was considered the upper limit of tolerability in non-cancerous cells.

No reduction in cell viability was observed in cells exposed to DMSO, confirming that the solvent used for compound preparation did not exert cytotoxic effects. Based on these findings, a concentration of 10 µg/mL 5-fluorouracil was selected for subsequent experiments.

#### 2.1.2. Cell Viability Following 24 h Exposure to Thymoquinone

**Figure 2:**
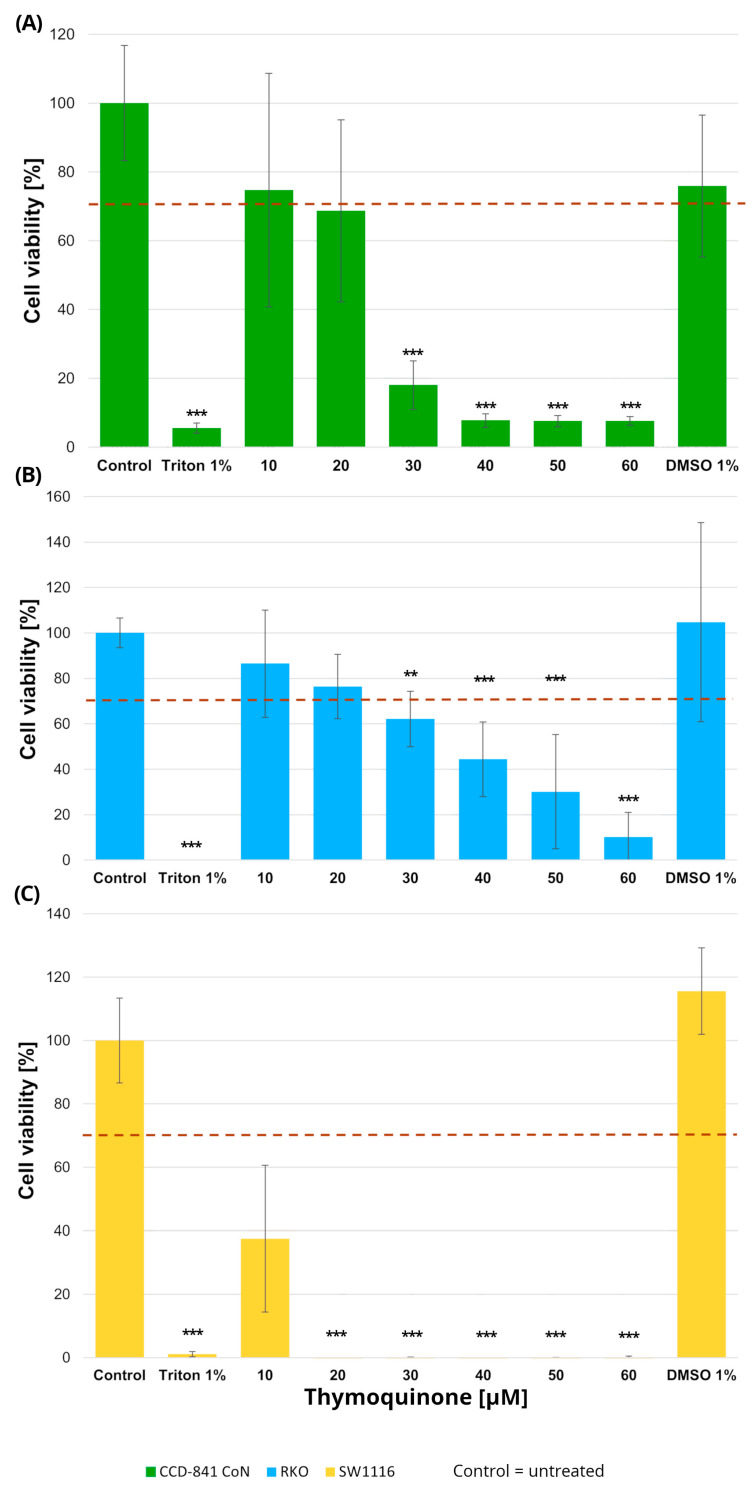
Cell viability of normal colon epithelial cells (CCD-841CoN; (**A**)) and colorectal cancer cell lines RKO (**B**) and SW1116 (**C**) following 24 h treatment with thymoquinone at increasing concentrations. Cell viability was assessed using the MTT assay and expressed as a percentage of the untreated control (set to 100%). Bars represent mean ± standard deviation (SD). The dashed horizontal line indicates the 70% viability threshold. Untreated cells served as the control, Triton X-100 (1%) was used as a positive cytotoxicity control, and DMSO (1%) served as the vehicle control corresponding to the highest solvent concentration applied. Statistical significance was evaluated using the Kruskal–Wallis one-way ANOVA followed by post hoc pairwise mean-rank comparisons; ** *p* < 0.01; *** *p* < 0.001 compared with the control. Three independent biological replicates were analyzed. Abbreviations: DMSO, dimethyl sulfoxide.

Normal colon epithelial cells and colorectal cancer cell lines were treated with increasing concentrations of thymoquinone (10–60 µM), resulting in distinct viability profiles across the examined cell types (Figure 2).

In RKO cells, thymoquinone induced a significant reduction in cell viability at concentrations of 30 µM and above. Specifically, cell viability decreased to 62 ± 12% at 30 µM (*p* = 0.004), 44 ± 16% at 40 µM (*p* = 0.000002), 30 ± 25% at 50 µM (*p* < 1 × 10^−6^), and 10 ± 10% at 60 µM (*p* < 1 × 10^−6^), with the most pronounced effects observed in the 40–60 µM range. SW1116 cells exhibited an even higher sensitivity to thymoquinone, with statistically significant decreases in viability observed already at 20 µM and persisting across higher concentrations. At concentrations ≥ 30 µM, cell viability was reduced to near-complete loss of viable cells, reaching values below 1% of control levels (*p* < 1 × 10^−6^), indicating a broader and more pronounced sensitivity to thymoquinone compared with RKO cells.

In the normal colon epithelial CCD-841CoN cell line, cell viability remained above the 70% cytotoxicity threshold only at 10 µM and 20 µM thymoquinone, whereas a significant reduction was observed from 30 µM onward, with viability decreasing to 18 ± 7% (*p* = 0.0001) and further declining at higher concentrations (40–60 µM; *p* < 1 × 10^−6^). Accordingly, 20 µM was identified as the highest concentration that maintained acceptable viability in non-cancerous cells.

No cytotoxic effects of the vehicle control (DMSO), used for thymoquinone preparation, were observed in any of the tested cell lines.

Based on these findings, a thymoquinone concentration of 20 µM was selected for subsequent experiments, as it produced measurable anticancer effects while preserving normal cell viability within the variability range around the 70% threshold.

#### 2.1.3. Cell Viability and Bliss Independence Analysis of the Thymoquinone and 5-Fluorouracil Combination

**Figure 3:**
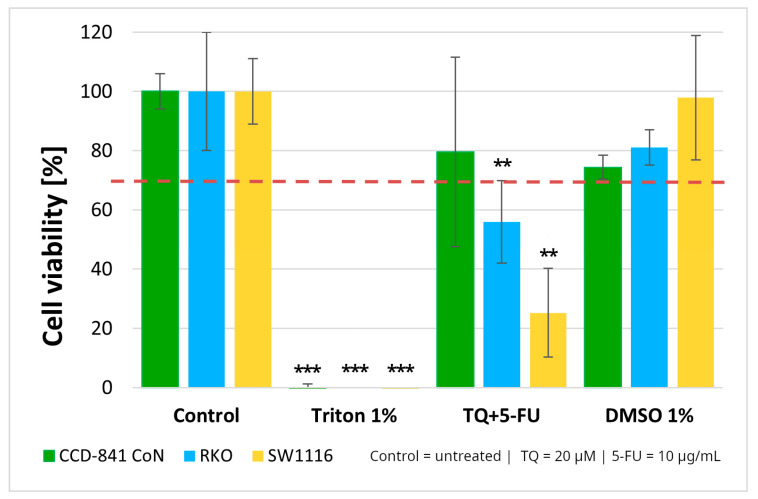
Cell viability of normal colon epithelial cells (CCD-841CoN) and colorectal cancer cell lines RKO and SW1116 following 24 h treatment with thymoquinone combined with 5-fluorouracil (TQ + 5-FU). Cell viability was assessed using the MTT assay and expressed relative to the untreated control (set to 100%). Bars represent mean ± standard deviation (SD). The dashed horizontal line indicates the 70% viability threshold. Untreated cells served as the control, Triton X-100 (1%) was used as a positive cytotoxicity control, and DMSO (1%) served as the vehicle control. Statistical significance was evaluated using the Kruskal–Wallis one-way ANOVA followed by post hoc pairwise mean-rank comparisons; ** *p* < 0.01; *** *p* < 0.001 compared with the control. Abbreviations: TQ, thymoquinone; DMSO, dimethyl sulfoxide.

Normal colon epithelial cells and colorectal cancer cell lines were treated with a combination of thymoquinone (20 µM) and 5-fluorouracil (10 µg/mL). The combined treatment resulted in statistically significant reductions in cell viability in both colorectal cancer cell lines, with viability decreasing to 56 ± 14% in RKO cells (*p* = 0.004) and 25 ± 15% in SW1116 cells (*p* = 0.003). In contrast, no cytotoxic effects were observed in the normal colon epithelial CCD-841CoN cells under the same treatment conditions (Figure 3).

These findings, together with the viability patterns observed for the single agents treatments, supported the selection of this concentration combination for subsequent mechanistic experiments. As 1% DMSO did not affect cell viability, the vehicle control was not included in further assays.

To further characterize the interaction between thymoquinone and 5-fluorouracil, a Bliss independence analysis was performed (Table 1).

In RKO cells, the observed inhibitory effect closely approximated the predicted Bliss value (Δ*Bliss* ≈ −0.09), indicating the absence of a synergistic interaction. In SW1116 cells, in which thymoquinone alone produced near-complete cytotoxicity, the combined treatment resulted in lower-than-expected inhibitory effect (Δ*Bliss* ≈ −0.25), consistent with an antagonistic interaction. Similarly, negative Δ*Bliss* values observed in CCD-841CoN cells (Δ*Bliss* ≈ −0.13) indicated no enhancement of cytotoxicity in normal epithelial cells. As only a single concentration pair was analyzed, the Bliss assessment provides a qualitative evaluation of drug interaction rather than a dose-dependent interaction profile.

### 2.2. DNA Fragmentation

**Figure 4:**
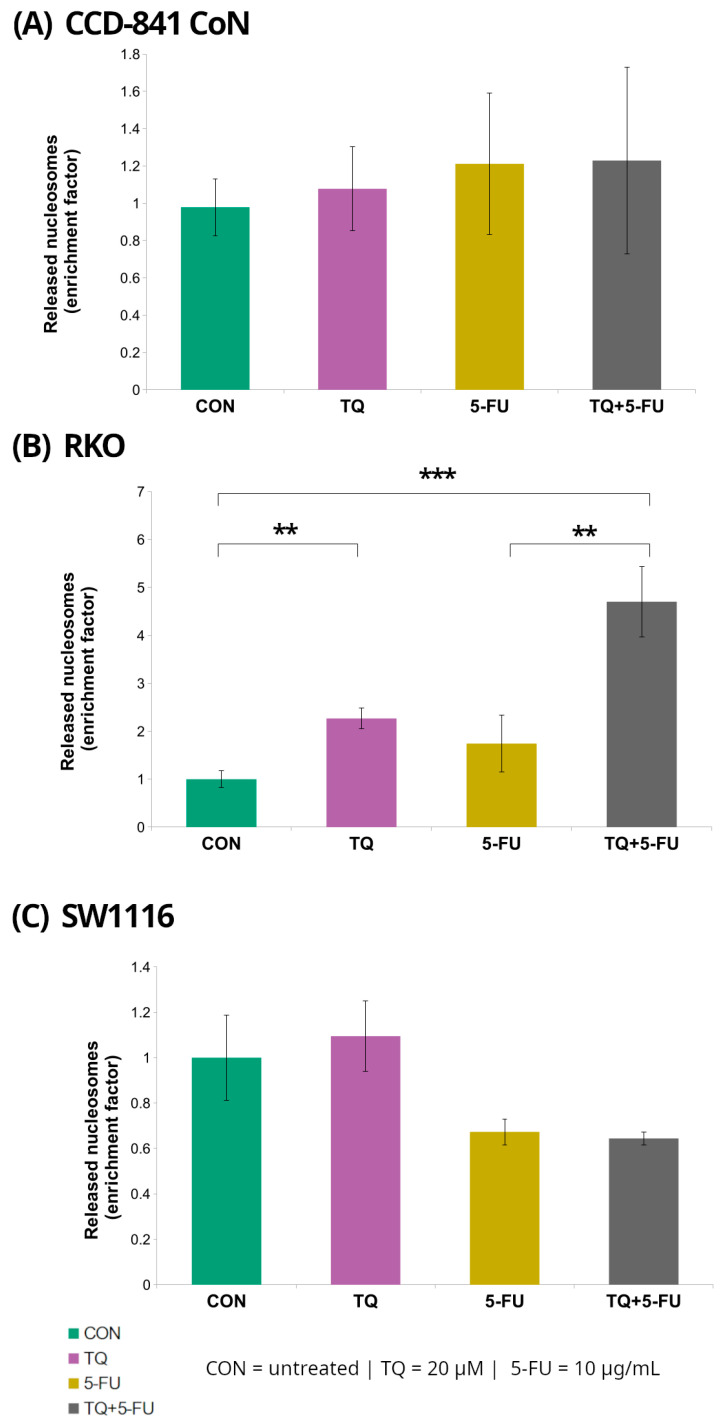
Enrichment of cytoplasmic mono- and oligonucleosomes in normal colon epithelial cells (CCD-841CoN; (**A**)) and colorectal cancer cell lines RKO (**B**) and SW1116 (**C**) following 24 h treatment with thymoquinone (TQ; 20 µM), 5-fluorouracil (5-FU; 10 µg/mL), or their combination. Nucleosome levels were quantified using the Cell Death Detection ELISA assay and expressed as an enrichment factor relative to the untreated control (set to 1.0). Bars represent mean ± standard deviation (SD). Statistical significance was assessed using the Kruskal–Wallis one-way ANOVA followed by post hoc pairwise mean-rank comparisons; statistically significant differences between the indicated treatment groups are shown by brackets (** *p* < 0.01; *** *p* < 0.001). Abbreviations: CON, control; TQ, thymoquinone; 5-FU, 5-fluorouracil.

Histone-associated DNA fragmentation following 24 h exposure to 5-fluorouracil, thymoquinone, or their combination was assessed using the Cell Death Detection ELISA assay in normal colon epithelial cells (CCD-841CoN) and colorectal cancer cell lines (RKO and SW1116) (Figure 4).

Among the examined cell lines, RKO cells exhibited the most pronounced response. Treatment with 20 µM thymoquinone resulted in a significant 2.3 ± 0.22-fold increase in the DNA fragmentation enrichment factor relative to untreated controls (*p* = 0.008). In contrast, combined treatment with thymoquinone and 5-fluorouracil produced a markedly stronger effect, yielding a 4.7 ± 0.74-fold increase (*p* = 0.000002). Notably, the enrichment factor observed following the combined treatment was also significantly higher than that detected after treatment with 5-fluorouracil alone (4.7 ± 0.74 vs. 1.74 ± 0.59; *p* = 0.008).

In contrast, no statistically significant changes in DNA fragmentation were detected in CCD-841CoN or SW1116 cells under any of the tested treatment conditions, indicating that the increase in nucleosomal DNA fragmentation was specific to RKO cells.

### 2.3. Caspase-3/7, Caspase-8, and Caspase-9 Activity

**Figure 5:**
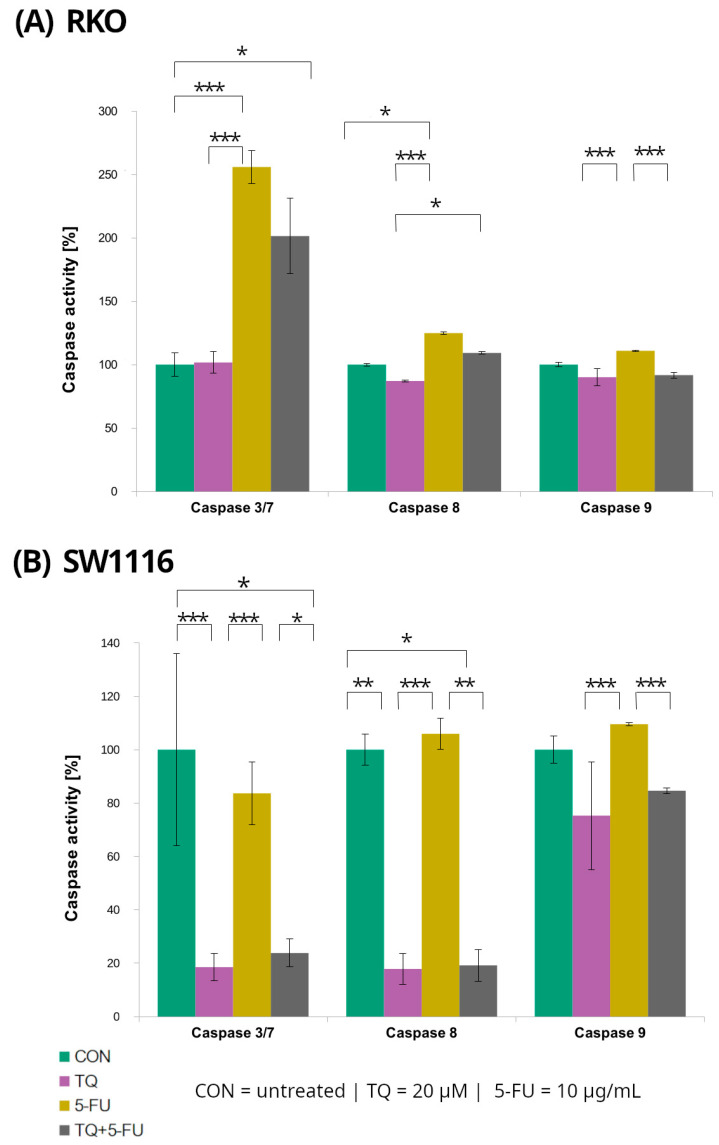
Caspase-3/7, caspase-8, and caspase-9 activity in RKO (**A**) and SW1116 (**B**) colorectal cancer cells following 24 h treatment with thymoquinone (TQ; 20 µM), 5-fluorouracil (5-FU; 10 µg/mL), or their combination. Caspase activity was measured using Caspase-Glo^®^ 3/7, Caspase-Glo^®^ 8, and Caspase-Glo^®^ 9 assay kits and expressed as a percentage of the untreated control (set to 100%). Bars represent mean ± standard deviation (SD). Statistical significance was evaluated using the Kruskal–Wallis one-way ANOVA followed by post hoc pairwise mean-rank comparisons; statistically significant differences between the indicated treatment groups are shown by brackets (* *p* < 0.05; ** *p* < 0.01; *** *p* < 0.001). Abbreviations: CON, control; TQ, thymoquinone; 5-FU, 5-fluorouracil.

Caspase-3/7, caspase-8, and caspase-9 activities were evaluated in RKO and SW1116 colorectal cancer cell lines following 24 h treatment with 5-fluorouracil, thymoquinone, or their combination using luminescent assays (Figure 5). Distinct response patterns were observed between the two cell lines.

In RKO cells, caspase-3/7 activity increased significantly following treatment with 5-fluorouracil and with the combined TQ + 5-FU regimen, reaching 256 ± 13% (*p* = 0.0001) and 201 ± 30% (*p* = 0.04) of control levels, respectively. In contrast, thymoquinone alone did not affect caspase-3/7 activity, which remained comparable to that of untreated control cells. Conversely, SW1116 cells exhibited a marked suppression of caspase-3/7 activity following thymoquinone treatment and combined TQ + 5-FU exposure, with activity levels reduced to 19 ± 5% (*p* = 0.0007) and 24 ± 5% (*p* = 0.022) of control values, respectively.

Caspase-8 activity displayed a similar cell line-dependent pattern. In SW1116 cells, caspase-8 activity was significantly reduced following thymoquinone treatment (18 ± 5%, *p* = 0.006) and combined treatment 19 ± 6%, *p* = 0.014), whereas 5-FU alonedid not significantly differ from the control levels. In RKO cells, a significant increase in caspase-8 activity was observed only after 5-FU treatment reaching 125 ± 1% of control (*p* = 0.016), while thymoquinone alone or in combination with 5-FU did not induce significant changes.

For caspase-9, variability among treatment groups was observed in both RKO and SW1116 cells; however, none of these differences reached statistical significance compared with the respective untreated controls.

### 2.4. Assessment of Cell Viability and Death Pathways by Flow Cytometry

**Figure 6:**
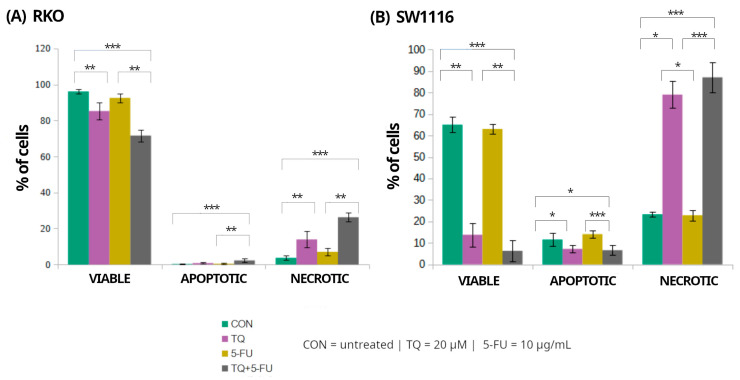
Flow cytometric assessment of cell viability and cell death in RKO and SW1116 colorectal cancer cells following 24 h exposure to thymoquinone (TQ; 20 µM), 5-fluorouracil (5-FU; 10 µg/mL), or their combination. Cells were stained using the Vybrant™ DyeCycle™ Violet/SYTOX™ AADvanced™ Apoptosis Kit and analyzed by flow cytometry. Quantitative distributions of viable, apoptotic, and necrotic cell populations are presented as bar graphs for RKO (**A**) and SW1116 (**B**). Data are shown as mean percentages ± standard deviation (SD). Statistical significance was evaluated using the Kruskal–Wallis one-way ANOVA followed by post hoc pairwise mean-rank comparisons; statistically significant differences between the indicated treatment groups are shown by brackets (* *p* < 0.05; ** *p* < 0.01; *** *p* < 0.001). Abbreviations: CON, untreated control; TQ, thymoquinone; 5-FU, 5-fluorouracil.

Flow cytometry was employed to assess the effects of 5-fluorouracil, thymoquinone, and their combination on the distribution of viable, apoptotic, and necrotic cell populations in RKO and SW1116 colorectal cancer cell lines after 24 h of treatment (Figure 6). Markedly distinct cell death profiles were observed between the two cell lines, indicating differential activation of death pathways in response to the applied treatments.

In RKO cells, exposure to thymoquinone alone or in combination with 5-fluorouracil resulted in a significant reduction in the proportion of viable cells compared with untreated controls, decreasing to 85 ± 5% (*p* = 0.009) and 72 ± 3% (*p* = 0.000004), respectively. These treatments were accompanied by a marked increase in the necrotic cell population reaching 14 ± 4% (*p* = 0.008) and 26 ± 3% (*p* = 0.000004), respectively. In contrast, apoptotic cells accounted for only a minor fraction of the total population, comprising 0.8 ± 0.5% (*p* > 0.05) and 2 ± 1% (*p* = 0.0004) following thymoquinone alone and combined treatment, respectively. Treatment with 5-fluorouracil alone did not significantly affect the distribution of viable, apoptotic, or necrotic cells.

In SW1116 cells, thymoquinone and the combined TQ + 5-FU treatment resulted in a pronounced loss of viability, with the proportion of viable cells decreasing to 14 ± 5% (*p* = 0.008) and 6 ± 5% (*p* = 0.0001), respectively, compared with untreated controls. This reduction in viability was accompanied by a substantial shift toward necrotic cell death, with necrotic cells accounting for 79 ± 6% (*p* = 0.023) and 87 ± 7% (*p* = 0.0006) of the total population following thymoquinone alone and combined treatment, respectively. Similarly to RKO cells, treatment with 5-fluorouracil alone did not significantly alter cell viability or the proportions of apoptotic and necrotic populations.

Overall, these results indicate that thymoquinone, administered alone or in combination with 5-fluorouracil, predominantly induced necrotic cell death in both colorectal cancer cell lines, with SW1116 cells exhibiting a more pronounced response than RKO cells. In contrast, 5-fluorouracil alone exerted minimal cytotoxic effects under the applied experimental conditions.

### 2.5. Transcriptional Activity of Cell Death-Regulating Genes

To gain insight into the molecular pathways underlying cellular responses to thymoquinone (20 µM), 5-fluorouracil (10 µg/mL), and their combination, the transcriptional activity of genes involved in the regulation of apoptosis and necroptosis was analyzed in normal colon epithelial cells (CCD-841CoN) and colorectal cancer cell lines (RKO and SW1116). The examined gene panel included initiator and executioner caspases (*CASP8*, *CASP9*, *CASP3*, and *CASP7*), intrinsic apoptosis regulators (*BAX* and *BCL2*), components of the extrinsic death receptor pathway (*FAS* and *FADD*), and key mediators of necroptosis (*RIPK1*, *RIPK3*, and *MLKL*). This comprehensive profiling enabled assessment of whether the observed changes in cell viability and cell death phenotypes were associated with transcriptional modulation across major programmed cell death pathways.

#### 2.5.1. Expression of Initiator Caspases (*CASP8* and *CASP9*)

**Figure 7:**
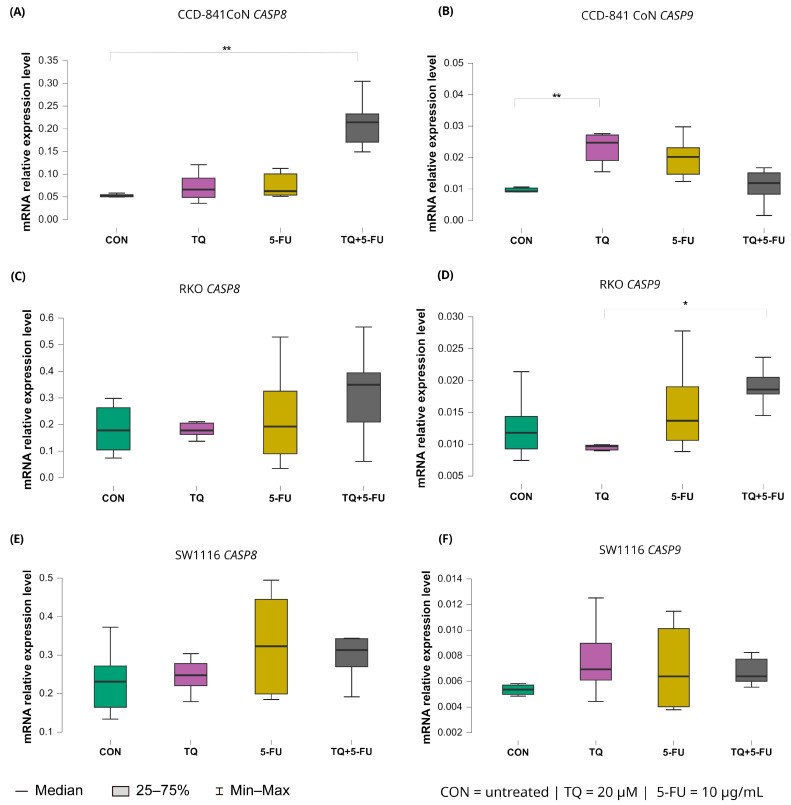
Expression levels of *CASP8* and *CASP9* in normal colon epithelial cells (CCD-841CoN) and colorectal cancer cell lines RKO and SW1116 following 24 h treatment with thymoquinone (TQ; 20 µM), 5-fluorouracil (5-FU; 10 µg/mL), or their combination. Panels (**A**,**B**) show *CASP8* and *CASP9* expression in CCD-841CoN cells, panels (**C**,**D**) depict expression in RKO cells, and panels (**E**,**F**) present expression in SW1116 cells. Box plots represent the interquartile range (IQR), with the horizontal line indicating the median and whiskers denoting values within 1.5× IQR. Statistical significance was assessed using the Kruskal–Wallis one-way ANOVA followed by post hoc pairwise mean-rank comparisons, statistically significant differences between groups are indicated by asterisks (* *p* < 0.05; ** *p* < 0.01). Data are derived from three biological replicates with three technical replicates per group. Abbreviations: CON, untreated control; TQ, thymoquinone; 5-FU, 5-fluorouracil.

Changes in the expression levels of the initiator caspases *CASP8* and *CASP9* following 24 h exposure to thymoquinone, 5-fluorouracil, or their combination are presented in Figure 7.

*CASP8* expression remained largely unchanged in both colorectal cancer cell lines, indicating a limited transcriptional response to any of the applied treatments. In contrast, CCD-841CoN cells exhibited a significant increase in *CASP8* expression exclusively following combined treatment with thymoquinone and 5-fluorouracil compared with untreated controls with median values of 0.214 (IQR: 0.171–0.233) and 0.052 (IQR: 0.050–0.055), respectively (*p* = 0.0017). This finding suggests a treatment-specific effect confined to non-malignant cells.

A distinct expression pattern was observed for *CASP9*. In CCD-841CoN cells, thymoquinone alone significantly upregulated *CASP9* expression relative to untreated controls as reflected by median values of 0.025 (IQR: 0.019–0.027) and 0.009 (IQR: 0.009–0.010), respectively (*p* = 0.0042). In RKO cells, *CASP9* expression differed significantly between thymoquinone monotherapy and the combined TQ + 5-FU treatment with median values of 0.010 (IQR: 0.010–0.010) and 0.019 (IQR: 0.018–0.021), respectively (*p* = 0.031), indicating treatment-dependent modulation. However, neither condition resulted in a clear upregulation relative to baseline levels. In SW1116 cells, *CASP9* expression remained unchanged across all tested treatment conditions.

#### 2.5.2. Expression of Executioner Caspases (*CASP3* and *CASP7*)

**Figure 8:**
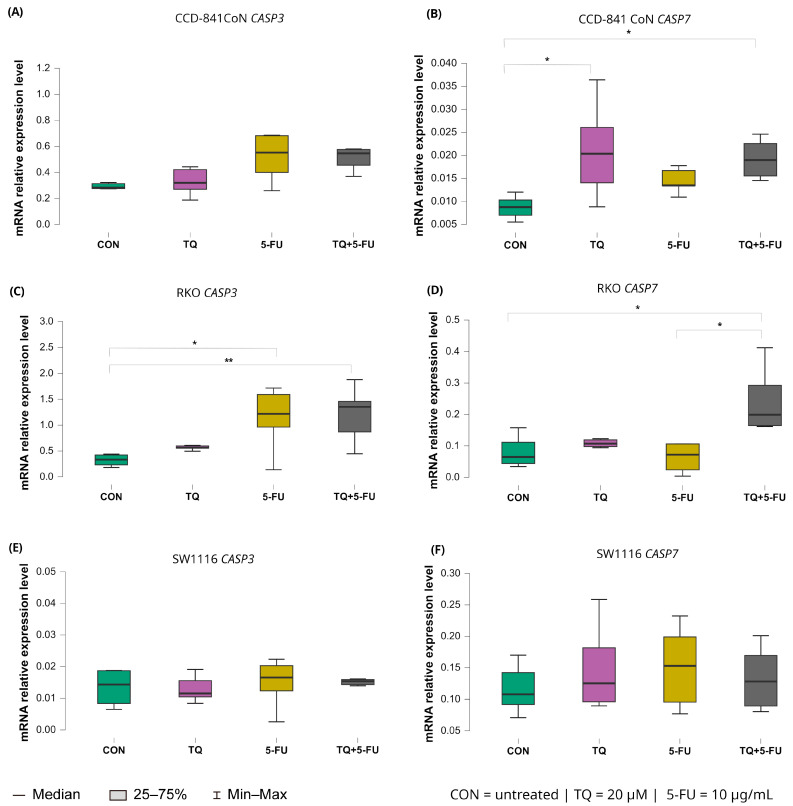
Expression levels of *CASP3* and *CASP7* in normal colon epithelial cells (CCD-841CoN) and colorectal cancer cell lines RKO and SW1116 following 24 h treatment with thymoquinone (TQ; 20 µM), 5-fluorouracil (5-FU; 10 µg/mL), or their combination. Panels (**A**,**B**) show *CASP3* and *CASP7* expression in CCD-841CoN cells, panels (**C**,**D**) present expression in RKO cells, and panels (**E**,**F**) depict expression in SW1116 cells. Box plots represent the interquartile range (IQR), with the horizontal line indicating the median and whiskers denoting values within 1.5× IQR. Statistical significance was assessed using the Kruskal–Wallis one-way ANOVA followed by post hoc pairwise mean-rank comparisons, statistically significant differences between groups are indicated by asterisks (* *p* < 0.05; ** *p* < 0.01). Data are derived from three biological replicates with three technical replicates per group. Abbreviations: CON, untreated control; TQ, thymoquinone; 5-FU, 5-fluorouracil.

The transcriptional responses of the executioner caspases *CASP3* and *CASP7* displayed cell line–specific patterns following 24 h exposure to thymoquinone, 5-fluorouracil, or their combination (Figure 8).

For *CASP3,* a significant increase in expression was observed exclusively in RKO cells. Both 5-fluorouracil alone (median: 1.218; IQR: 0.962–1.592) and the combined TQ + 5-FU treatment (median: 1.354; IQR: 0.869–1.459) induced significant upregulation relative to untreated controls (*p* = 0.026 and *p* = 0.0065, respectively). No significant changes in *CASP3* expression were detected in CCD-841CoN or SW1116 cells under any treatment condition.

In contrast, CASP7 exhibited broader treatment responsiveness. In RKO cells, *CASP7* expression increased significantly following the combined TQ + 5-FU treatment, exceeding both untreated controls and the 5-FU monotherapy group, with median values of 0.199 (IQR: 0.165–0.293), 0.065 (IQR: 0.044–0.111; *p* = 0.026), and 0.072 (IQR: 0.024–0.106; *p* = 0.029), respectively.

In CCD-841CoN cells, thymoquinone alone and the combined treatment also resulted in significant upregulation of *CASP7* expression compared with untreated controls, with median values of 0.020 (IQR: 0.014–0.026; *p* = 0.029) and 0.019 (IQR: 0.016–0.023; *p* = 0.017), respectively, versus 0.009 (IQR: 0.007–0.010) in control cells. No significant modulation of *CASP7* expression was observed in SW1116 cells across the tested conditions.

#### 2.5.3. Intrinsic Pathway Regulators: *BAX* and *BCL2*

**Figure 9:**
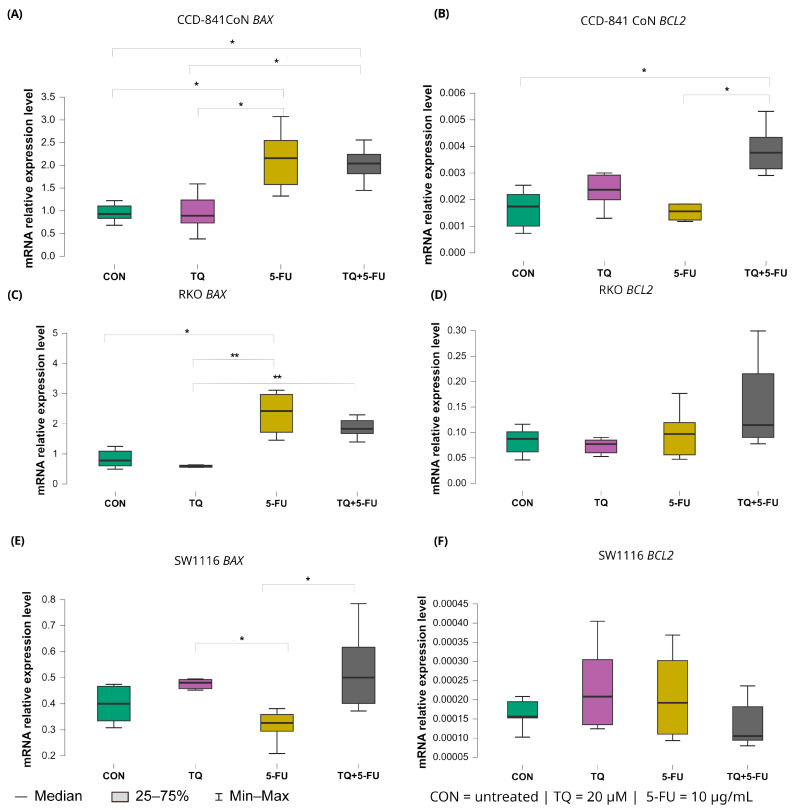
Expression levels of *BAX* and *BCL2* in normal colon epithelial cells (CCD-841CoN) and colorectal cancer cell lines RKO and SW1116 following 24 h treatment with thymoquinone (TQ; 20 µM), 5-fluorouracil (5-FU; 10 µg/mL), or their combination. Panels (**A**,**B**) show *BAX* and *BCL2* expression in CCD-841CoN cells, panels (**C**,**D**) depict expression in RKO cells, and panels (**E**,**F**) present expression in SW1116 cells. Box plots represent the interquartile range (IQR), with the horizontal line indicating the median and whiskers denoting values within 1.5× IQR. Statistical significance was assessed using the Kruskal–Wallis one-way ANOVA followed by post hoc pairwise mean-rank comparisons, statistically significant differences between groups are indicated by asterisks (* *p* < 0.05; ** *p* < 0.01). Data are derived from three biological replicates with three technical replicates per group. Abbreviations: CON, untreated control; TQ, thymoquinone; 5-FU, 5-fluorouracil.

Treatment-dependent modulation of intrinsic apoptosis regulators *BAX* and *BCL2* is presented in Figure 9.

In RKO cells, 5-fluorouracil markedly increased *BAX* expression relative to untreated controls with median values 2.423 (IQR: 1.719–2.975) and 0.782 (IQR: 0.603–1.094), respectively (*p* = 0.040). Moreover, *BAX* expression in the 5-FU-treated group was significantly higher than that observed following thymoquinone monotherapy (median: 2.423 [IQR: 1.719–2.975] vs. 0.588 [IQR: 0.558–0.627]; *p* = 0.0025). The combined TQ + 5-FU treatment also resulted in higher *BAX* expression compared with TQ alone, with median values of 1.834 (IQR: 1.630–2.196) and 0.588 (IQR: 0.558–0.627), respectively (*p* = 0.009).

In SW1116 cells, both thymoquinone alone and the combined treatment produced *BAX* expression levels that differed significantly from those observed following 5-FU monotherapy. Median values were 0.480 (IQR: 0.314–0.473; *p* = 0.042) for thymoquinone, 0.500 (IQR: 0.401–0.617; *p* = 0.026) for the combined treatment, and 0.305 (IQR: 0.284–0.359) for 5-FU, indicating treatment-specific transcriptional responses in this chemoresistant cell line.

In normal CCD-841CoN cells, *BAX* expression was significantly elevated following both 5-FU monotherapy and the combined TQ + 5-FU treatment compared with untreated controls. Median values were 2.156 (IQR: 1.576–2.547) and 2.039 (IQR: 1.814–2.424), respectively (*p* = 0.024 and *p* = 0.031). Both conditions also differed significantly from the thymoquinone-only group (median: 0.929 [IQR: 0.834–1.105]; *p* = 0.027 and 0.891 [IQR: 0.730–1.237]; *p* = 0.035, respectively).

In contrast, expression of the anti-apoptotic gene *BCL2* remained largely unchanged in the colorectal cancer cell lines. A significant difference was detected only in CCD-841CoN cells exposed to the combined TQ + 5-FU treatment, with median value of 0.004 (IQR: 0.003–0.004), which differed significantly from untreated controls and from 5-FU alone (both median: 0.002 [IQR: 0.001–0.002]; *p* = 0.022 and *p* = 0.042, respectively). No significant modulation of *BCL2* expression was observed in either RKO or SW1116 cells under any treatment condition.

#### 2.5.4. Extrinsic Death Receptor Pathway: *FAS* and *FADD*

**Figure 10:**
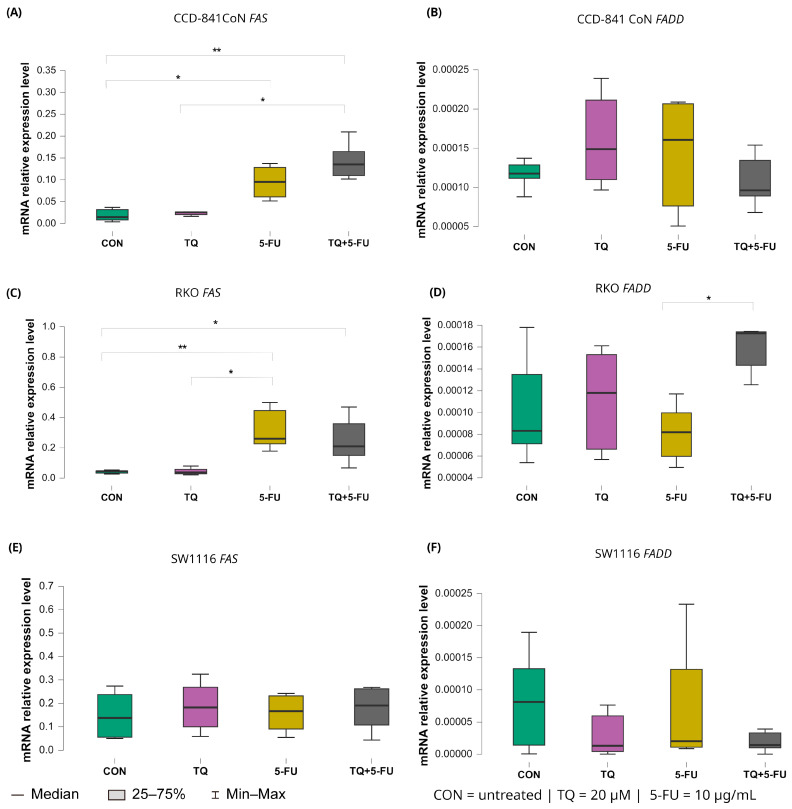
Expression levels of *FAS* and *FADD* in normal colon epithelial cells (CCD-841CoN) and colorectal cancer cell lines RKO and SW1116 following 24 h treatment with thymoquinone (TQ; 20 µM), 5-fluorouracil (5-FU; 10 µg/mL), or their combination. Panels (**A**,**B**) show *FAS* and *FADD* expression in CCD-841CoN cells, panels (**C**,**D**) depict expression in RKO cells, and panels (**E**,**F**) present expression in SW1116 cells. Box plots represent the interquartile range (IQR), with the horizontal line indicating the median and whiskers denoting values within 1.5× IQR. Statistical significance was assessed using the Kruskal–Wallis one-way ANOVA followed by post hoc pairwise mean-rank comparisons statistically significant differences between groups are indicated by asterisks (* *p* < 0.05; ** *p* < 0.01). Data are derived from three biological replicates with three technical replicates per group. Abbreviations: CON, untreated control; TQ, thymoquinone; 5-FU, 5-fluorouracil.

Changes in the expression levels of the extrinsic apoptosis regulators *FAS* and *FADD* following treatment with thymoquinone, 5-fluorouracil, or their combination are presented in Figure 10.

In CCD-841CoN cells, baseline *FAS* expression in untreated controls was low (median: 0.017, IQR: 0.014–0.033). *FAS* expression increased significantly following exposure to 5-fluorouracil (median: 0.095, IQR: 0.061–0.128; *p* = 0.022 vs. control) or the combined TQ + 5-FU treatment (median: 0.135, IQR: 0.110–0.165; *p* = 0.002 vs. control). Moreover, the combined regimen resulted in significantly higher *FAS* expression than thymoquinone alone (median: 0.022, IQR: 0.017–0.026; *p* = 0.017).

A similar pattern was observed in RKO cells. Both 5-FU (median 0.248, IQR 0.230–0.437; *p* = 0.0061 vs. control) and the combined TQ + 5-FU treatment (median 0.220, IQR 0.173–0.317; *p* = 0.045 vs. control) significantly upregulated *FAS* expression relative to untreated controls (median 0.050, IQR 0.047–0.053). In addition, *FAS* expression following 5-FU treatment differed significantly from that observed after TQ monotherapy (median: 0.048, IQR: 0.047–0.058; *p* = 0.011). In contrast, *FAS* expression in SW1116 cells remained unchanged across all treatment groups, indicating limited transcriptional responsiveness of the extrinsic death receptor pathway in this cell line.

For *FADD*, transcriptional modulation was minimal. A significant difference was detected only in RKO cells treated with the combined TQ + 5-FU regimen compared with 5-FU alone, with median values of 0.00017 (IQR: 0.00014–0.00017) and 0.000082 (IQR: 0.000060–0.000098), respectively (*p* = 0.029). No significant changes in *FADD* expression were observed in CCD-841CoN or SW1116 cells under any treatment condition.

#### 2.5.5. Necroptosis Pathway: *RIPK1*, *RIPK3*, and *MLKL*

**Figure 11:**
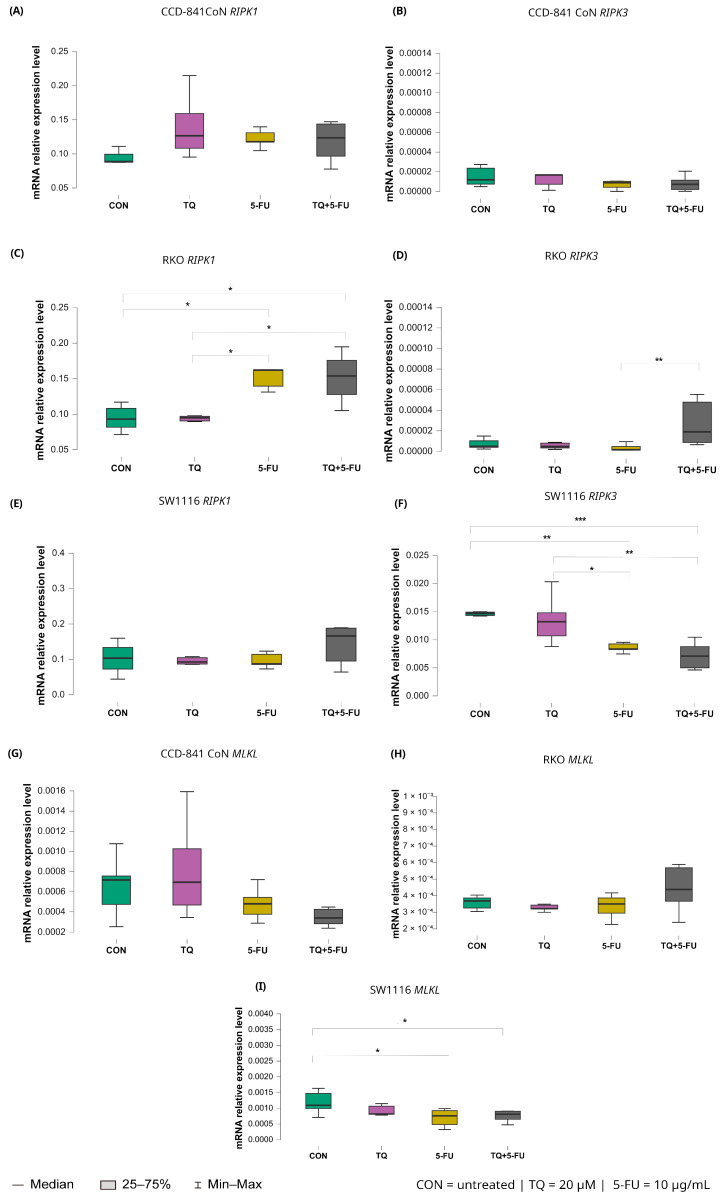
Expression levels of *RIPK1*, *RIPK3*, and *MLKL* in normal colon epithelial cells (CCD-841CoN) and colorectal cancer cell lines RKO and SW1116 following 24 h treatment with thymoquinone (TQ; 20 µM), 5-fluorouracil (5-FU; 10 µg/mL), or their combination. Panels (**A**–**C**) show *RIPK1*, *RIPK3*, and *MLKL* expression in CCD-841CoN cells, panels (**D**–**F**) depict expression in RKO cells, and panels (**G**–**I**) present expression in SW1116 cells. Box plots represent the interquartile range (IQR), with the horizontal line indicating the median and whiskers denoting values within 1.5× IQR. Statistical significance was assessed using the Kruskal–Wallis one-way ANOVA followed by post hoc pairwise mean-rank comparisons, statistically significant differences between groups are indicated by asterisks (* *p* < 0.05; ** *p* < 0.01; *** *p* < 0.001). Data are derived from three biological replicates with three technical replicates per group. Abbreviations: CON, untreated control; TQ, thymoquinone; 5-FU, 5-fluorouracil.

Changes in the expression levels of necroptosis-related genes following exposure to thymoquinone, 5-fluorouracil, or their combination are presented in Figure 11.

Among the analyzed targets, *RIPK1* exhibited treatment-dependent modulation exclusively in RKO cells. Both 5-fluorouracil monotherapy and the combined TQ + 5-FU treatment significantly increased *RIPK1* expression compared with untreated controls, with median values of 0.162 (IQR: 0.139–0.163; *p* = 0.026) and 0.154 (IQR: 0.128–0.176; *p* = 0.042), respectively, versus 0.093 (IQR: 0.082–0.108) in control cells. In addition, *RIPK1* expression following 5-FU-containing treatments differed significantly from that observed after thymoquinone monotherapy (*p* = 0.015), with median values of 0.162 (IQR: 0.139–0.163) and 0.095 (IQR: 0.090–0.098), respectively.

For *RIPK3*, divergent responses were observed among the analyzed cell lines. In RKO cells, the combined TQ + 5-FU treatment significantly upregulated *RIPK3* expression compared with 5-FU alone, with median values of 0.0000232 (IQR: 0.0000132–0.000042) and 0.00000163 (IQR: 0.00000148–0.00000182), respectively (*p* = 0.0011). In contrast, SW1116 cells exhibited reduced *RIPK3* expression following both 5-FU monotherapy and the combined treatment, with median values of 0.008 (IQR: 0.008–0.009) and 0.007 (IQR: 0.005–0.009), respectively.

These expression levels were markedly lower than those observed in untreated control cells (5-FU vs. control, *p* = 0.0041; TQ + 5-FU vs. control, *p* = 0.00038), which showed a median expression value of 0.015 with an IQR of 0.014–0.015. Similarly, expression levels were significantly lower than those detected in thymoquinone-treated cells, which exhibited a median value of 0.013 (IQR: 0.011–0.015) (5-FU vs. TQ, *p* = 0.029; TQ + 5-FU vs. TQ, *p* = 0.0019).

Expression of *MLKL* remained unchanged in CCD-841CoN and RKO cells across all treatment conditions. In contrast, SW1116 cells exhibited a significant decrease in *MLKL* expression following both 5-fluorouracil monotherapy and the combined TQ + 5-FU treatment compared with untreated controls. Median values were 0.000762 (IQR: 0.000483–0.000925; *p* = 0.023) and 0.000811 (IQR: 0.000649–0.000907; *p* = 0.049), respectively, versus 0.001 (IQR: 0.00099–0.001) in control cells. These findings indicate a cell line-specific transcriptional response of necroptosis-related genes.

## 3. Discussion

Colorectal cancer remains a major global health burden due to its high incidence and mortality, with a particularly concerning rise in early-onset cases reported in developed countries [2,3]. Despite advances in surgical and systemic treatment strategies, fluoropyrimidine-based regimens, most notably those centered on 5-fluorouracil, continue to form the backbone of colorectal cancer therapy. However, their clinical efficacy is frequently compromised by intrinsic or acquired chemoresistance and treatment-associated toxicities that adversely affect patient quality of life [9,11,54].

In recent years, natural bioactive compounds have attracted increasing attention as modulators of anticancer responses owing to their multitarget mechanisms of action and generally favorable safety profiles [25,26,27]. Among these compounds, thymoquinone, the principal bioactive constituent of *Nigella sativa* seeds, has demonstrated anti-inflammatory, antioxidant, and anticancer properties in numerous experimental models [29,30,32,34]. Nevertheless, the mechanisms underlying TQ-induced cytotoxicity appear to be highly context dependent and remain incompletely understood in colorectal cancer.

In the present study, we systematically evaluated the effects of thymoquinone alone and in combination with 5-fluorouracil in two colorectal cancer cell lines with distinct molecular characteristics, RKO and SW111, alongside normal colon epithelial cells (CCD-841CoN). By integrating cell viability assays, DNA fragmentation analysis, caspase activity measurements, flow cytometry-based phenotyping, and transcriptional profiling of apoptosis- and necroptosis-related genes, we aimed to elucidate cell line-dependent death pathways triggered by TQ.

### 3.1. Selective Cytotoxicity of Thymoquinone Toward Colorectal Cancer Cells

Our findings demonstrate that both colorectal cancer cell lines were susceptible to thymoquinone-induced cytotoxicity, whereas normal colon epithelial cells remained largely unaffected under the applied conditions. Notably, although combined treatment with TQ and 5-FU reduced cancer cell viability in both models, the mode of cell death differed fundamentally between RKO and SW1116 cells, underscoring the importance of cellular context in shaping death execution pathways.

### 3.2. Context-Dependent Apoptotic Response of RKO Cells

RKO cells, which exhibit microsatellite instability and retain wild-type p53, are known to be highly prone to apoptotic signaling [47,48,55,56]. Consistent with this phenotype, TQ treatment induced pronounced DNA fragmentation in RKO cells, with an even greater effect observed following combined TQ + 5-FU exposure. These findings were accompanied by increased caspase-3/7 activity and transcriptional upregulation of key components of the extrinsic apoptotic pathway, including *CASP8*, *FAS*, and *BAX*, indicating engagement of apoptosis-related signaling cascades.

Comparable pro-apoptotic effects of TQ, alone or in combination with 5-FU, have been reported in other colorectal cancer models. Studies in HCT116 and SW480 cells demonstrated enhanced apoptosis, increased DNA damage, and reduced proliferation following co-treatment with TQ and fluoropyrimidines [42,57]. Together, these observations support the notion that TQ can facilitate apoptotic signaling in apoptosis-competent colorectal cancer cells.

Interestingly, flow cytometric analysis revealed a substantial increase in necrotic cell populations in RKO cells following TQ and TQ + 5-FU treatment. This apparent discrepancy between apoptotic markers and necrotic morphology likely reflects secondary necrosis, a terminal phase of apoptosis that occurs when apoptotic bodies are not efficiently cleared.

Apoptosis is a temporally regulated process in which early activation of caspases and executioner pathways precedes morphological changes and loss of plasma membrane integrity. During the early and intermediate stages of apoptosis, membrane integrity is largely preserved, whereas membrane permeabilization typically occurs at later stages, particularly in in vitro systems lacking efficient clearance of apoptotic bodies, ultimately resulting in secondary necrosis [58,59]. Given that all analyses were performed at the 24 h time point, it is plausible that caspase activation and apoptotic signaling occurred at earlier stages of treatment, whereas loss of plasma membrane integrity became detectable at later stages, manifesting as secondary necrosis in flow cytometry. Importantly, the absence of transcriptional activation of necroptosis-related genes, including *RIPK3* and *MLKL*, further supports the interpretation that regulated necroptosis was not engaged in RKO cells. Given the prominent necrotic and secondary necrotic cell death observed in both SW1116 and RKO cells, future studies incorporating intracellular reactive oxygen species (ROS) measurements may provide additional insight into the contribution of oxidative stress to thymoquinone-induced cytotoxicity. However, it should be noted that the use of a single 24 h time point represents a limitation of this study, as it does not allow precise temporal tracking of early apoptotic events versus late-stage secondary necrotic processes.

### 3.3. Caspase-Independent Cell Death in Chemoresistant SW1116 Cells

In contrast, SW1116 cells characterized by chromosomal instability, stemness-associated features, and poor responsiveness to 5-FU [49,60,61] exhibited a fundamentally different response to TQ. In this model, TQ alone or in combination with 5-FU induced marked loss of viability accompanied by extensive necrotic cell death, as determined by flow cytometry. Importantly, this cytotoxicity occurred in the absence of caspase activation, nucleosomal DNA fragmentation, or transcriptional upregulation of key apoptotic genes, indicating a caspase-independent mode of cell death.

The lack of *RIPK3* and *MLKL* induction in SW1116 cells further excludes classical necroptosis as the dominant mechanism. Instead, the observed phenotype is consistent with non-programmed necrotic cell death, potentially driven by severe cellular stress, mitochondrial dysfunction, membrane destabilization, or oxidative damage. Similar caspase-independent TQ-induced cytotoxicity has been reported in chemoresistant colorectal cancer and other tumor models, including CPT-11-resistant LoVo cells, diffuse large B-cell lymphoma, and glioblastoma, where TQ triggered alternative death programs involving mitochondrial dysfunction, autophagy, or lysosomal pathways [41,62,63].

### 3.4. Selective Resistance of Normal Colon Epithelial Cells to Thymoquinone

Importantly, normal CCD-841CoN cells remained largely resistant to TQ-induced cytotoxicity. Despite modest transcriptional modulation of several apoptosis-related genes, including *BAX*, *CASP7*, and *CASP9*, no loss of viability or DNA fragmentation was observed. This pattern suggests activation of a sublethal stress response rather than execution of programmed cell death, consistent with previous reports demonstrating the selective anticancer activity and favorable safety profile of thymoquinone in non-malignant cells [35,36,37,38,39].

### 3.5. Lack of Synergistic Interaction Between Thymoquinone and 5-Fluorouracil

Bliss independence analysis further revealed that the interaction between thymoquinone and 5-fluorouracil was non-synergistic across all tested cell lines. In RKO cells, the combination exhibited additive to mildly antagonistic behavior, while in SW1116 cells, where TQ alone produced near-complete cytotoxicity, the addition of 5-FU resulted in a clearly antagonistic interaction. These findings indicate that the contribution of 5-FU to the observed cytotoxic effects is limited under the tested conditions and that TQ acts primarily as an independent modulator of cell death pathways rather than as a pharmacological sensitizer.

### 3.6. Strengths and Limitations of the Study

Several limitations of the present study should be acknowledged. The experiments were conducted at a single concentration and time point, restricting evaluation of dose- and time-dependent effects. In addition, morphological changes were not documented by inverted light microscopy, and no photomicrographs were collected to visually support the viability and cell death assays. Furthermore, although necrotic and secondary necrotic cell death was observed, intracellular reactive oxygen species production was not assessed, precluding direct evaluation of oxidative stress-related mechanisms.

Moreover, mechanistic conclusions are primarily based on mRNA expression and functional readouts, without direct protein-level validation or the use of pathway-specific inhibitors. Although three independent biological replicates were used for RT-qPCR analysis in accordance with commonly accepted experimental standards, increasing the number of biological replicates would further enhance statistical power and data robustness. Future studies incorporating caspase inhibition, modulation of oxidative stress, detailed morphological analysis, extended concentration matrices, and multiple time points will be essential to further delineate the molecular determinants of TQ-induced cell death.

Despite these limitations, the present study has several notable strengths. The use of two colorectal cancer cell lines representing distinct molecular subtypes enabled a comparative analysis of context-dependent cellular responses to thymoquinone. The integration of complementary experimental approaches, including viability assays, flow cytometry-based cell death profiling, and transcriptional analysis, provided a multidimensional assessment of thymoquinone-induced cytotoxicity. Importantly, the inclusion of normal colon epithelial cells allowed evaluation of selective anticancer effects, underscoring the favorable safety profile of thymoquinone under the applied experimental conditions.

### 3.7. Concluding Remarks

In summary, this study demonstrates that thymoquinone triggers distinct, cell line-dependent death pathways in colorectal cancer cells. While apoptosis-associated mechanisms predominate in RKO cells, TQ induces caspase-independent necrotic cell death in the chemoresistant SW1116 model, without affecting normal colon epithelial cells. These findings highlight the context-dependent nature of thymoquinone cytotoxicity and underscore its potential as a selective modulator of cell death pathways in colorectal cancer.

## 4. Materials and Methods

### 4.1. Cell Lines and Culture Conditions

The colorectal cancer cell lines RKO and SW1116 (CRL-2577 and CCL-233, respectively) and the normal colon epithelial cell line CCD-841CoN (CRL-1790) were obtained from the American Type Culture Collection (ATCC; Manassas, VA, USA). Cells were routinely cultured at 37 °C in a humidified atmosphere containing 5% CO_2_ using a Direct Heat CO_2_ Incubator (Thermo Scientific, Waltham, MA, USA).

RKO and CCD-841CoN cells were maintained in Eagle’s Minimum Essential Medium (EMEM), whereas SW1116 cells were cultured in Dulbecco’s Modified Eagle Medium (DMEM). All culture media were supplemented with 10% fetal bovine serum (FBS; EuroClone, Milan, Italy) and 50 µg/mL gentamicin (BioWhittaker, Lonza, Basel, Switzerland) [64].

Cells were subcultured in standard tissue culture flasks (Thermo Scientific, Waltham, MA, USA) and used for experiments within six passages to ensure phenotypic stability and reproducibility. Upon reaching approximately 80% confluence, cells were detached using 0.25% trypsin–EDTA (Sigma-Aldrich, Merck, St. Louis, MO, USA). Both adherent and floating cells were collected when required to avoid loss of apoptotic or non-adherent populations. All cell lines were routinely monitored for morphology and viability, and mycoplasma-free status was confirmed prior to experimentation.

### 4.2. Cell Treatment Protocol

RKO, SW1116, and CCD-841CoN cells were seeded into 96-well culture plates (Thermo Scientific, Waltham, MA, USA) at a density of 1 × 10^4^ cells per well and allowed to attach for 48 h. Following stabilization, cells were treated with thymoquinone, 5-fluorouracil, or their combination.

Thymoquinone (Sigma-Aldrich, St. Louis, MO, USA) was applied at concentrations ranging from 10 to 60 µM, and 5-fluorouracil (Sigma-Aldrich, St. Louis, MO, USA) at concentrations of 10–100 µg/mL (corresponding to approximately 76.8–768 µM). Dose ranges were selected based on preliminary viability screening and previously published data to enable consistent evaluation of dose-dependent responses [39,45,65,66,67]. For combination treatments, both compounds were added simultaneously at the indicated concentrations.

Stock solutions were prepared in dimethyl sulfoxide (Sigma-Aldrich, St. Louis, MO, USA), with the final solvent concentration maintained below 1% (*v*/*v*), which also served as the vehicle control. All working solutions were sterile-filtered through 0.2 µm syringe filters (Sartorius, Göttingen, Germany) immediately before use. After 24 h of treatment, cells were processed for downstream assays.

### 4.3. MTT Cell Viability Assay

Cell viability was evaluated using the MTT assay (3-(4,5-dimethylthiazol-2-yl)-2,5-diphenyltetrazolium bromide; Sigma-Aldrich, St. Louis, MO, USA), which is based on the reduction of MTT to insoluble formazan crystals by metabolically active cells [68,69].

After 24 h of exposure to TQ, 5-FU, or their combination, the culture medium was removed and replaced with MTT solution (1 mg/mL in phosphate-buffered saline, PBS). Cells were incubated for 3 h at 37 °C in the dark to allow for formazan formation. Subsequently, the MTT solution was discarded, and the formazan crystals were solubilized in DMSO, as previously described [65].

Absorbance was measured at 570 nm with a reference wavelength of 650 nm using a BioTek Epoch microplate spectrophotometer (Agilent Technologies, Santa Clara, CA, USA). Cells treated with 1% DMSO served as the solvent control, while 1% Triton X-100 (Sigma-Aldrich, St. Louis, MO, USA) was used as a positive control for cytotoxicity. Cell viability was expressed as a percentage relative to untreated control cells. A viability threshold of 70% was adopted to define cytotoxic effects.

### 4.4. Drug Interaction Analysis Based on the Bliss Independence Model

The Bliss independence model was applied to evaluate the interaction between 5-fluorouracil (compound A) and thymoquinone (compound B) [70]. Cell viability data obtained from the MTT assay were converted into effect values using the following transformation:

E=1−V

where *V* represents viability normalized to the untreated control. Viability values slightly below 0% were set to 0 (corresponding to complete loss of viability) to avoid artifacts related to assay variability.

The expected combined effect was calculated according to the Bliss independence equation:

$$E_{Bliss}=E_{A}+E_{B}-E_{A}E_{B}$$

where *E_A_* and *E_B_* represent the individual effects of 5-FU and TQ, respectively. The observed combined effect (*E_Obs_*) was then compared with the expected Bliss value (*E_Bliss_*) to classify drug interactions as synergistic (*E_Obs_* > *E_Bliss_*), antagonistic (*E_Obs_* < *E_Bliss_*), or additive (*E_Obs_* ≈ *E_Bliss_*).

The magnitude of interaction was quantified as the Bliss excess:

$$∆Bliss=\;E_{Obs}-\;E_{Bliss}$$

### 4.5. DNA Fragmentation Assay

DNA fragmentation was evaluated using the Cell Death Detection ELISA kit (Sigma-Aldrich, St. Louis, MO, USA) following 24 h exposure to the tested compounds. This assay is based on a quantitative sandwich ELISA employing monoclonal antibodies specific for histones and DNA, enabling detection of cytoplasmic mono- and oligonucleosomes generated during apoptosis-associated internucleosomal DNA cleavage, which precedes the loss of plasma membrane integrity [71].

Cells were cultured and treated as described above, and all subsequent procedures were performed according to the manufacturer’s instructions. Absorbance was measured at 405 nm using a BioTek Epoch microplate spectrophotometer (Agilent Technologies, Santa Clara, CA, USA). DNA fragmentation was expressed as an enrichment factor calculated as the ratio of absorbance in treated samples to that of untreated control cells, reflecting the fold increase in apoptotic nucleosome release.

### 4.6. Assessment of Caspase Activity

Caspase activity was assessed exclusively in the colorectal cancer cell lines RKO and SW1116, as preliminary experiments indicated that treatment with thymoquinone or 5-fluorouracil, either alone or in combination, did not induce detectable cytotoxicity or apoptosis in CCD-841CoN cells under the experimental conditions applied.

Activities of caspase-3/7, caspase-8, and caspase-9 were measured using Caspase-Glo^®^ luminescent assay kits (Promega, Madison, WI, USA). These assays are based on a homogeneous add-and-measure format in which the addition of a single reagent results in cell lysis and release of a luminogenic substrate selectively cleaved by the respective caspase. Substrate cleavage generates a stable glow-type luminescent signal produced by luciferase, with signal intensity directly proportional to enzymatic activity in the sample [72].

Cells were cultured and treated as described above, and all procedures were performed according to the manufacturer’s instructions. Luminescence was recorded using a microplate luminometer (Promega, Madison, WI, USA). Caspase activity was expressed as a percentage of the luminescent signal relative to untreated control cells.

### 4.7. Flow Cytometry-Based Cell Death Analysis

Flow cytometry was used to analyze cell death-associated changes in the colorectal cancer cell lines RKO and SW1116, as initial viability and DNA fragmentation assays did not indicate apoptotic responses in CCD-841CoN cells under the experimental conditions applied. Following 24 h exposure to thymoquinone, 5-fluorouracil, or their combination, both adherent and floating cells were collected to avoid loss of non-adherent dying cell populations.

Flow cytometric staining was performed using the Vybrant™ DyeCycle™ Violet/SYTOX™ AADvanced™ Apoptosis Kit (Thermo Fisher Scientific, Waltham, MA, USA) according to the protocol described by Madej et al. [73]. Briefly, cells were resuspended in assay buffer and incubated with DyeCycle™ Violet to assess DNA content and chromatin condensation, followed by staining with SYTOX™ AADvanced™ to evaluate plasma membrane integrity.

Data acquisition was performed using a FACSAria™ II flow cytometer (BD Biosciences, San Jose, CA, USA). A minimum of 10,000 events per sample were recorded. Data were analyzed using BD FACSDiva software (v6.1.2). Cells were classified as viable, apoptotic, or necrotic based on fluorescence characteristics. All experiments were performed in eight independent biological replicates.

### 4.8. Quantitative Real-Time Polymerase Chain Reaction (RT-qPCR) Assay

Quantitative real-time RT-qPCR was performed to determine the expression levels of selected genes. Relative mRNA expression was calculated using the 2^−ΔCt^ method, with Ct values of target genes normalized to the geometric mean of the reference housekeeping genes GAPDH and TBP to obtain ΔCt values [74]. Target and reference genes were amplified in parallel to ensure reliable comparative analysis.

RT-qPCR reactions were carried out using the SensiFAST™ SYBR^®^ Hi-ROX One-Step Kit (Bioline, London, UK). This assay is based on SYBR Green chemistry, in which fluorescence increases upon binding to double-stranded DNA, enabling real-time monitoring of amplicon accumulation.

Primer sequences used in this study are listed in Table 2.

Amplification was performed using a LightCycler^®^ 480 System (Roche, Basel, Switzerland) in accordance with the manufacturer’s instructions. The thermal cycling protocol included a reverse transcription step at 45 °C for 10 min, followed by initial denaturation at 95 °C for 2 min and 45 amplification cycles. Each cycle consisted of denaturation at 95 °C for 5 s, annealing at 60 °C for 10 s, and extension at 72 °C for 5 s. A melting-curve analysis was performed at the end of each run to verify amplification specificity. This approach ensured accurate and reproducible quantification of gene expression. RT-qPCR experiments were performed using three independent biological replicates, each analyzed in triplicate as technical replicates, in accordance with accepted methodological guidelines [75].

### 4.9. Statistical Analysis

Statistical analyses were performed using STATISTICA software (version 13.3; TIBCO Software Inc., Palo Alto, CA, USA), with statistical significance defined as *p* < 0.05. Quantitative RT-qPCR results are presented as medians with interquartile ranges (IQR), including minimum and maximum values, due to non-normal data distribution and the limited number of biological replicates [65,73]. RT-qPCR data were visualized using JASP software (version 0.19.1.0; University of Amsterdam, Amsterdam, The Netherlands). All other quantitative data are expressed as mean ± standard deviation (SD), as these datasets showed approximately symmetrical distributions and are commonly reported in this format in similar experimental studies. Data visualization for these datasets was performed using Microsoft Excel (Microsoft Corporation, Redmond, WA, USA) and LibreOffice Calc (The Document Foundation, Berlin, Germany).

Normality of data distribution was assessed using the Shapiro–Wilk test, while homogeneity of variances was evaluated using Levene’s test. As these assumptions were not met for the majority of datasets, non-parametric statistical methods were applied [76]. Accordingly, group comparisons were performed using the Kruskal–Wallis one-way analysis of variance, followed by post hoc pairwise comparisons based on mean ranks.

Statistical outliers were identified using the interquartile range (IQR) method and excluded from the final analyses to prevent distortion of group-level comparisons. All statistical tests and exclusion criteria were applied consistently across datasets to ensure analytical rigor and reproducibility.

## 5. Conclusions

In conclusion, this study demonstrates that thymoquinone induces pronounced but fundamentally different cell death responses in colorectal cancer cells depending on their molecular background. In apoptosis-competent RKO cells, thymoquinone predominantly engaged apoptosis-associated signaling, accompanied by DNA fragmentation and caspase activation, with secondary necrotic features observed at later stages. In contrast, in the chemoresistant SW1116 model, thymoquinone triggered extensive caspase-independent necrotic cell death without activation of apoptotic or necroptotic pathways. Importantly, normal colon epithelial cells remained largely unaffected, indicating selective cytotoxicity toward malignant cells. Although combined treatment with 5-fluorouracil reduced cancer cell viability, Bliss analysis revealed non-synergistic interactions, highlighting thymoquinone as a modulator of cell death execution rather than a classical chemosensitizer. Collectively, these findings underscore the context-dependent nature of thymoquinone-induced cytotoxicity and support its further investigation as a bioactive compound capable of differentially targeting apoptosis-sensitive and apoptosis-resistant colorectal cancer phenotypes.

## Figures and Tables

**Table 1:** Bliss independence analysis of the interaction between thymoquinone (TQ) and 5-fluorouracil (5-FU) in normal colon epithelial cells (CCD-841CoN) and colorectal cancer cell lines (RKO, SW1116). *E_TQ_* and *E_5-FU_* represent the effects of the individual compounds, *E_Obs_* denotes the observed combined effect, and *E_Bliss_* indicates the expected effect according to the Bliss independence model. Δ*Bliss* was calculated as Δ*Bliss* = *E_Obs_* − *E_Bliss_*. Negative Δ*Bliss* values indicate non-synergistic or antagonistic interactions.

Δ*Bliss*	*E_Bliss_* (Expected)	*E_Obs_* (TQ + 5-FU)	*E_5-FU_*	*E_TQ_*	TQ + 5-FU Viability	TQ Viability	5-FU Viability	Cell Line
−0.13	0.331	0.20	0.03	0.31	80%	69%	97%	CCD 841CoN
−0.09	0.529	0.44	0.38	0.24	56%	76%	62%	RKO
−0.25	1.00	0.75	0.16	1	25%	0%	84%	SW1116

**Table 2:** Primer sequences used for quantitative real-time RT-qPCR analysis.

Reverse Primer (5′ → 3′)	Forward Primer (5′ → 3′)	Gene Symbol
TGCACACAGGTCTTCTTC	CTAGACCTCTTCTCCATGC	*FADD*
AAGATTTCATCCACAGAGGG	GTGAAGAATGTGAAGACTGG	*MLKL*
ACAGTTTTTCCAGTGCTTTC	TGATAATACCACTAGTCTGACG	*RIPK1*
GTTGTATATGTTAACGAGCGG	AACTTTCAGAAACCAGATGC	*RIPK3*
CCACCCTGGTCTTGGATCCAGCCC	CCTGTGCACCAAGGTGCCGGAACT	*BAX*
CGAACAAAGCCTTTAACTTG	TAAATCCTGAAACAGTGGC	*FAS*
TTGCTGCATCGACATCTGTA	TGGATTATCCTGAGATGGGTTT	*CASP3*
CATGGCTTAAGAGGATGCAG	ACTGCTCTTGTGCCAAGATG	*CASP7*
TTTCACCGAAACAGCATTAG	CTCTACTTTCCCAGGTTTTG	*CASP9*
ATTTGGAGATTTCCTCTTGC	CTACAGGGTCATGCTCTATC	*CASP8*
GAAGATGGTGATGGGATTC	GAAGGTGAAGGTCGGAGT	*GAPDH*
GAAGTCCAAGAACTTAGCTG	GCCAAGAGTGAAGAACAG	*TBP*
CACTTGATTCTGGTGTTTCC	AACATCACAGAGGAAGTAGAC	*BCL2*

## Data Availability

The original contributions presented in this study are included in the article. Further inquiries can be directed to the corresponding author.

## Abbreviations

The following abbreviations are used in this manuscript:

5-FU	5-fluorouracil
CIN	Chromosomal instability
CRC	Colorectal cancer
DMSO	Dimethyl sulfoxide
DMEM	Dulbecco’s Modified Eagle Medium
EMEM	Eagle’s Minimum Essential Medium
FBS	Fetal bovine serum
IQR	Interquartile range
MSI	Microsatellite instability
PBS	Phosphate-buffered saline
ROS	Reactive oxygen species
SD	Standard deviation
TQ	Thymoquinone
